# Liebig's Physiology Applied in the Treatment of Functional Derangement and Organic Disease. With Observations upon Hahnemann's Practice.—Part I. The Heart, Lungs, Stomach, Glands, Joints, Bones, &c., with Cases, Showing the Advantages of Modern Science over Former Methods in the Treatment of Disease

**Published:** 1846-07

**Authors:** 


### Art. XXIII.

*Liebig's Physiology applied in the Treatment of Functional Derangement and Organic Disease. With Observations upon Hahnemann's Practice.* —Part I. *The Heart, Lungs, Stomach, Glands, Joints, Bones, &c., with Cases, showing the Advantages of Modern Science over Former Methods in the Treatment of Disease.* By John Leeson, Practitioner of Medicine, Divisional Surgeon of the Metropolitan Police Force, &c. &c.— *London*, 1846. 8vo, pp. 219.

Among the penalties, which are attached to any post of distinction, is the impudent assumption of a name which has acquired eminence or notoriety of almost any kind, to force into public notice the productions of those pretenders to science, whose ignorance and venality are about upon a par, their sole idea of it being—what it will fetch in the market. Thus, we have seen Liebig's Manure, and Liebig's Beer advertised like Locock's Pulmonic Wafers, the Bug-destroyers to her Majesty, the London University Wine-vaults, the Great Western Shoe-shop, and the like. And we now have Liebig's Medicine for the Million advertised, not by a professed empiric, but, we are sorry to say, by an M.R.C.S, a Fellow of the Royal Medical and Chirurgical Society, and a Divisional Surgeon to the Metropolitan Police-Force.

We shall not inflict upon our readers any extended demonstration of the justice of the comparison we have made; for we think that a few short extracts will make it apparent, that, so far as his literary labours are concerned, Mr. Leeson may be regarded as the legitimate type of those *flunkeys*—in Carlyle phrase—who don the livery of a great man, and then strut about with airs of arrogance which they think conformable to his dignity, betraying their ignorance of everything that constitutes true gentility in every word and movement.

Let our readers scrutinize the following brief quotations (the italics in which are our own), and say if we have judged too harshly.

"The theories propounded by Liebig, although not altogether new in many instances, are nevertheless put forward *under appearances of greater perspicuity than was accomplished by any other philosopher.*" (p. 24.)

"There can be no other way of explaining the laws of life and sustenance with anything like certainty, than by chemical analyses; as the resulting phenomena determine without much ambiguity the nature of the one and the manner of the other." (p. 24.)

What a profound conception is here manifested, of the nature of the relation between chemistry and vitality?

"Heat is *generated out af the union* of carbon and oxygen, and also out of hydrogen and oxygen; *nutrition from the food, out of which are* produced reparation and excretion." (p. 35.)

"The *action of oxygen* upon the living body is *a demand for food or for death.* The food *discloses all the elements* ,by which the destructive influence of the oxygen is neutralized, and every part and tissue of the system maintained." (p. 26.)

"The *gases* derived from the food, and *by which animal life is protected,* are *carbon,* hydrogen, and nitrogen,—they act in all the chemical combinations with the oxygen of inspiration, the human body itself instinctively regulating all their

*movements and adaptations* in such a manner, as, *in some instances,* to be beyond the comprehension of our ablest philosophers." (p. 27.)

"However, the alcoholic solutions may approximate in their amount of carbon and hydrogen to fats and oils, the difference of composition in each class is yet sufficient to maintain the most distinctive characters between them . . . . . The *rugged cares of life* are said to vanish before the influence of the alcoholic solutions; while the *fatty ones,* in small proportions, often produce nausea, sickness, and biliary derangements." (p. 30.)

Here we have a glimpse of a new and hitherto unexplored region in the wide domain of sublunary sorrow. "O reader, if that thou canst read," didst thou ever before read of the *adipose cares of life*? Perhaps not: but doubtless we all have witnessed such, with their "nausea, sickness, and biliary derangements," in the portly Aldermen and jolly Falstaffs of our corporation.

One of his most apposite references to the great master, whose pupil he desires to be accounted, is the following:

"Liebig wisely says that 'the deprivation of food soon puts a stop to all manifestations of vitality.' It would be difficult to dispute the soundness of this aphorism, when we can so readily discover in ourselves the depressive effects of even a temporary suspension of the ordinary means of nutrition." (p. 36.)

Wisely, indeed!—almost as wisely as if our own Tuson had spoken. Difficult to dispute!—so difficult, that the fact was known, we will venture to say, almost as soon as there were men on the earth to know it; and has continued to be a matter of the most familiar experience ever since. We scarcely think that Professor Liebig, although it might suit his argument to remind his readers of a fact so trite, ever dreamed of being cited as the author of the "aphorism." And we suspect he would scarcely own the following, although the statements profess to be but expositions of his doctrines:

"Animal and vegetable fibrine, albumen, and caseine, or the proteinaceous compounds, must all be resolved into their original elements, before the system will admit them to become parts of itself." (p. 37.)

"The urine consists principally of water, which enters the stomach as food and drink; after which it is taken into the circulation, where it becomes impregnated with the oxygen of inspiration, together with the various gases or vapours which are mixed or mechanically suspended in the atmosphere with which we might be surrounded; from thence it is transmitted to the kidneys for secretion into the bladder, from which it is expelled as necessity dictates." (p. 61.)

"Large quantities of the *ammoniacal gases* are taken in by the inspiratory process; and after having traversed through the circulatory system, are exhaled by the skin, as well as passed in the urine with little alteration, or combined with other gases, and with them form those ammoniacal salts which often appear in the urinary excretions. Large quantities of *carbon* are known to be constantly floating in a London atmosphere; and while it is the business of the system to expire carbon and oxygen, the same system is constantly taking in carbon, which is as often expelled through some or other of the excretory channels, and particularly in the urine under some or other of the *ammoniacal* combinations." (p. 63.)

Save me from my friends, may well be Liebig's exclamation, if he should chance to meet with such perversions of his meaning as are contained in the foregoing extracts.

So much as to the correctness of the exposition, which Mr. Leeson gives, of the *principles* of Liebig. The motive which dictates this enunciation of his *practice*, will at once appear from the following extract, which relates to no slight disease, but to nothing else than *consumption!*

"This view of the subject has often led me to the best method of treating this fearful disease, and with the most wonderful success. Cases for illustration I consider to be needless, as [the] soundness of the principles must appear obvious to the simplest understanding," (p. 98.)

There seems no other disease, among those to which allusion is made in this treatise, in which Mr. Leeson's practice does not claim equal success; and the question, therefore, naturally presents itself—Do Mr. Leeson's patients *ever* die? We have carefully scrutinized his account of his treatment; and whilst we have seen not a single idea worth anything, that is not already familiar to the mind of every well-educated practitioner, we observe continual omissions and errors, in regard to points as well understood as those just now cited. Consequently, the assumption of superiority, evident throughout the work, is so far from being justified by its contents, that Mr. Leeson's worst enemies—if enemies he has—could scarcely desire a more complete exposure of his ignorance and arrogance than he has here volunteered to present to the public. We denounce his book as disgraceful to any "Practitioner of Medicine," of whatsoever rank or name, and experience a sort of horror at the thought of its title making it be sought after and read by some of Liebig's learned countrymen. What a dainty dish of British medical literature and science to set before the stranger!

